# Transcriptomics Integrated With Metabolomics Reveal the Effects of Ultraviolet-B Radiation on Flavonoid Biosynthesis in Antarctic Moss

**DOI:** 10.3389/fpls.2021.788377

**Published:** 2021-12-08

**Authors:** Shenghao Liu, Shuo Fang, Chenlin Liu, Linlin Zhao, Bailin Cong, Zhaohui Zhang

**Affiliations:** ^1^Key Laboratory of Marine Ecology and Environment Science, First Institute of Oceanography, Natural Resources Ministry, Qingdao, China; ^2^Marine Ecology and Environmental Science Laboratory, Pilot National Laboratory for Marine Science and Technology, Qingdao, China

**Keywords:** abiotic stress, bryophytes, flavonoids, metabolome, transcriptome, ultraviolet-B radiation

## Abstract

Bryophytes are the dominant vegetation in the Antarctic continent. They have suffered more unpleasant ultraviolet radiation due to the Antarctic ozone layer destruction. However, it remains unclear about the molecular mechanism of Antarctic moss acclimation to UV-B light. Here, the transcriptomics and metabolomics approaches were conducted to uncover transcriptional and metabolic profiling of the Antarctic moss *Leptobryum pyriforme* under UV-B radiation. Totally, 67,290 unigenes with N_50_ length of 2,055 bp were assembled. Of them, 1,594 unigenes were significantly up-regulated and 3353 unigenes were markedly down-regulated under UV-B radiation. These differentially expressed genes (DEGs) involved in UV-B signaling, flavonoid biosynthesis, ROS scavenging, and DNA repair. In addition, a total of 531 metabolites were detected, while flavonoids and anthocyanins accounted for 10.36% of the total compounds. There were 49 upregulated metabolites and 41 downregulated metabolites under UV-B radiation. Flavonoids were the most significantly changed metabolites. qPCR analysis showed that UVR8-COP1-HY5 signaling pathway genes and photolyase genes (i.e., *LpUVR3*, *LpPHR1*, and *LpDPL*) were significantly up-regulated under UV-B light. In addition, the expression levels of JA signaling pathway-related genes (i.e., *OPR* and *JAZ*) and flavonoid biosynthesis-related genes were also significantly increased under UV-B radiation. The integrative data analysis showed that UVR8-mediated signaling, jasmonate signaling, flavonoid biosynthesis pathway and DNA repair system might contribute to *L. pyriforme* acclimating to UV-B radiation. Therefore, these findings present a novel knowledge for understanding the adaption of Antarctic moss to polar environments and provide a foundation for assessing the impact of global climate change on Antarctic land plants.

## Introduction

Ultraviolet-B radiation (280–315 nm) is an inherent part of sunlight. Increased UV-B light have been observed on the earth’s surface since the 1980s and 1990s due to depletion of stratospheric ozone layer, which results from increases of chlorofluorocarbons in the atmosphere (Hossaini et al., 2017; Neale et al., 2021). In addition to ozone effects, the UV-B light on the Earth’s surface is also affected by clouds, aerosols, reflectivity of the Earth’s surface and solar activity (Bais et al., 2019). Since the mid-1990s, the emission of ozone depleting substances (ODS) in the atmosphere has gradually decreased and the concentration has been declining, due to the efficient implementation of the Montreal Protocol and its amendments (Mckenzie et al., 2011). Currently, UV-B radiation are only slightly stronger than in 1980 (increases less than ∼5%) at mid-latitudes, but increases are enormous at high and polar latitudes where ozone depletion is increasing markedly (Mckenzie et al., 2011). In future, changes of UV-B radiation at middle and low latitudes will possibly be dominated by concentration of aerosols, while levels of UV-B radiation at high latitudes will be affected by stratospheric ozone recovery, cloud layer and surface reflectivity (Bais et al., 2019). In Antarctica, due to the positively anticipated recovery of ozone layer, the erythemal UV-B irradiance will be reduced up to 40% in the spring of 2100 (Bais et al., 2019). However, according to NASA’s report, the Antarctic ozone hole reached a peak of about 24 × 10^6^ km^2^ in early October 2020 and had extended to most areas of the Antarctic continent. The area of the Antarctic ozone hole was higher than the average in the past decade and the return of Antarctic ozone to pre-1980 levels could be substantially delayed (Hossaini et al., 2017).

The amount of UV-B light received at Earth’s surface greatly influences the terrestrial and aquatic ecosystems. High UV-B levels, larger than 1 μmol m^–2^ s^–2^ UV-B, is usually an environmental stress and cause the damage of their DNA, inducing cell to form pyrimidine dimer (Fuentes-León et al., 2020). Apart from DNA damage, high UV-B irradiance also causes membrane changes and protein crosslinking, and generates reactive oxygen species (ROS), as well as reduces the photosynthetic capacity and plant productivity (Núñez-Pons et al., 2018). However, low intensity of UV-B light can act as a growth signal mediating the plant photomorphogenesis (Kataria et al., 2014; Palma et al., 2020). Plants have evolved a variety of adaptive responses to UV-B light through morphological changes including cotyledon curling and hypocotyl growth inhibition (Dotto and Casati, 2017), and by biochemical changes including the accumulation of secondary metabolites such as flavonoids, anthocyanins, terpenoids, phenols, alkaloids, and beta-carotene (Ghasemi et al., 2019; Palma et al., 2020).

Plants are autotrophic sessile organisms and UVR8 (UV Resistance Locus 8) is the only characterized UV-B specific photoreceptor. Plants perceive UV-B light through the photoreceptor UVR8 and CONSTITUTIVE PHOTOMORPHOGENESIS1 (COP1) signaling pathway (Podolec et al., 2021). Upon UV-B light, UVR8 protein competitively binds to COP1 at the substrate binding site, inhibiting E3 ubiquitin ligase activity of COP1 and activating ELONGATED HYPOCOTYL5 (HY5) transcription factor (Tilbrook et al., 2016). The UVR8-COP1-HY5 form the main signaling components mediating the UV-B light signal transduction. In addition, UVR8 can also bind to a different set of transcription factors and directly inhibit their DNA binding. UVR8 can be inactivated by two WD40-repeat proteins RUP1 and RUP2, which provides negative feedback regulation (Podolec et al., 2021). The most current knowledge obtained from *Arabidopsis* showed that UVR8-mediated UV-B responses are accumulation of flavonols and anthocyanins, inhibition of hypocotyl growth, and changes in gene expression or protein accumulation (Yin and Ulm, 2017; Podolec et al., 2021). The UVR8-mediated signaling for inducing flavonoid accumulation was also conserved in the liverwort *Marchantia polymorpha* (Bowman et al., 2017; Kondou et al., 2019). Interestingly, UVR8-mediated signal transduction against UV-B radiation were also demonstrated in the green alga *Chlamydomonas reinhardtii* (Tilbrook et al., 2016). Although algae can generate purple phenolic pigments under abiotic stresses, UVB-induced flavonoid accumulation hasn’t been identified in algae (Davies et al., 2020).

Plants in the terrestrial ecosystems of the Antarctic continent are undergoing higher UV-B radiation of 3.4–6.2 mW/cm^2^ (Bao et al., 2018). These strong UV-B irradiation seriously restricts the growth and distribution of Antarctic terrestrial plants. Mosses and lichens are the dominant vegetation in the Antarctic ice free regions. The Antarctic field experiments demonstrate that chlorophyll contents were reduced in moss (*Bryum argenteum*) and lichen (*Umbilicaria aprina*) by continuing UV-B radiation, whereas the contents of UV-B absorbing compounds are increased (Singh and Singh, 2014). Several phenylpropanoids were found to function as protective barrier that increase resistance to UV radiation of moss *Ceratodon purpureus* in Antarctica (Clarke and Robinson, 2008). Meanwhile, flavonoids and carotenoids extracted from three Antarctic species [i.e., *Polytrichum juniperinum* Hedw, *Colobanthus quitensis* (Kunth) Bartl, and *Deschampsia antarctica* Desv] demonstrated the characteristics of UV-absorbing compounds, protecting cells and activating the DNA damage repair process (Pereira et al., 2009). It seems that these basal land plants can fight against the UV-B radiation through synthesizing antioxidants including UV-B-absorbing pigments, flavonoids and anthocyanins, functioning as the effective damage repair systems (Singh et al., 2011). However, anthocyanin compounds were not detected in the model plant *Physcomitrella patens* (Wolf et al., 2010). Currently, there are still limited about systematic and in-depth studies on the regulation of flavonoid biosynthesis and stress response in these basal land plants.

Here, the transcriptomics and metabolomics approaches were conducted to uncover transcriptional profiling of the Antarctic moss *Leptobryum pyriforme* under UV-B radiation. A total of 4947 differential expressed genes (DEGs) and 90 significantly changed metabolites (SCMs) were detected. Among them, flavonoids were one of the most significantly changed metabolites. An integrative data analysis demonstrated that UVR8-mediated signal signaling, Jasmonate signaling, flavonoid biosynthesis pathway and DNA repair system might play a critical role in the adaptation of the Antarctic moss *L. pyriforme* to UV-B radiation. Interestingly, our evidence confirmed that anthocyanins compounds were present in the Antarctic moss *L. pyriforme*.

## Experimental Procedures

### Plant Samples and Ultraviolet-B Radiation Treatments

**FIGURE 1:**
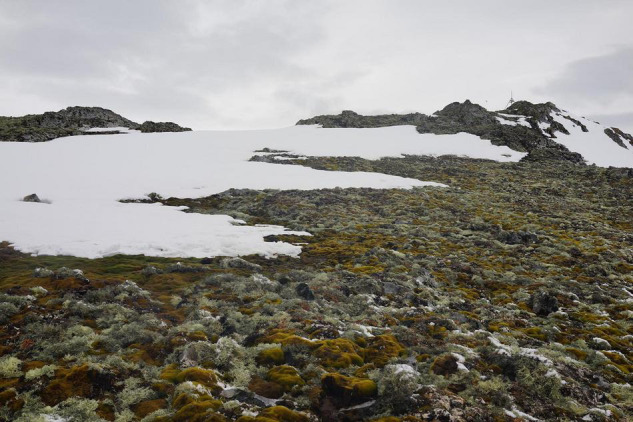
The field picture of Antarctic moss sampling site.

The moss samples were collected from the Fildes Peninsula of Antarctica (S62°12.851′, W58°56.253′; Figure 1). The moss *L. pyriforme* was separated and purified from mixed growth samples. They were cultivated on a soil mixture of Pindstrup substrate (Pindstrup Mosebrug A/S, Ryomgaard, Denmark) and local soil (ratio 1:1) at 16°C, 50 μmol photons⋅m^–2^⋅s^–1^ light with a 16-h-light/8-h-dark photoperiod. Seedlings were covered by transparent plastic film to keep moisture. Under this condition, the UV light is 0.09 mW/cm^2^ that produced by the Philips T8 TLD36W/54-765 fluorescent tubes.

Two Philips TL20W/01RS narrowband UV-B tubes were used for UV-B treatment as described previously (Wolf et al., 2010). The average level of UV-B light was 0.30 mW/cm^2^, which was measured by a UV-340A Ultraviolet Light Meter (Lutron Electronic Enterprise, Taiwan). Two Philips T8 TLD36W/54-765 fluorescent tubes were used to supplement the white light field and the photosynthetically active radiation was 13.5 μmol photons⋅m^–2^⋅s^–1^ (1000 lux) which was measured by a LX-101A Light Meter (Lutron Electronic Enterprise). Two-month-old plants were firstly acclimated to a low-white light field (13.5 μmol photons⋅m^–2^⋅s^–1^) for 48 h. Seedlings were then treated with 0.30 mW/cm^2^ UV-B light for 1.5 h, which were used for transcriptome sequencing. Seedlings treated with 0.30 mW/cm^2^ UV-B light for 5 days were used for LC-MS/MS analysis. The plants without UV-B radiation were collected and used as control group. The plants collected from different flowerpots were divided into three biological replicates. The green gametophytes were collected and rapidly frozen in liquid nitrogen, and stored at −80°C.

### Measurement of Total Antioxidant Capacity, Proline, Total Chlorophyll and Flavonoid Contents

Mosses were treated with UV-B light as described above. After 6 h or 72 h of UV-B radiation, the moss gametophytes were cut off and ground in liquid nitrogen. The extraction and reaction buffer were obtained from commercial kits (Nanjing Jiancheng Bioengineering Institute, Nanjing, China). 0.1 g of sample powder was used for each biochemical determining according to the kit instructions. Briefly, total antioxidant capacity was measured by ferric-reducing antioxidant power (FRAP) method. Proline quantitation was achievable by reaction with ninhydrin. Total chlorophylls were dissolved in organic solvent and detected on spectrophotometer according to the Lambert-Beer’s Law. Total flavonoids content was determined by ultraviolet-visible spectrophotometry at wavelengths of 325 nm. The experiments were repeated three times.

### RNA Isolation and Transcriptome Sequencing

Transcriptome sequencing were conducted following the standard procedure (Zhang et al., 2019). Briefly, 2 g of moss gametophyte samples were ground into power in liquid nitrogen. Total RNA was extracted by using Trizol reagent (Invitrogen, United States). RNA integrity was detected by Agilent 2100 Bioanalyzer (Agilent Technologies, United States), while RNA quality was analyzed by 1% agarose gel electrophoresis. mRNA was isolated from total RNA by oligo (dT) magnetic beads (New England Biolabs, United States). The enriched mRNA was broken into short fragments. cDNA fragments were synthesized after reverse transcription reaction. cDNA libraries were produced from appropriately fragmented cDNA using NEBNext^®^ Ultra™ RNA Library Prep Kit (New England Biolabs). The qualities of cDNA libraries of three UV-B groups and three control groups were detected by the Agilent Bioanalyzer 2100. Finally, the libraries were sequenced on an Illumina Hiseq 2500 platform.

### Sequence Assembly, Functional Annotation and Differentially Expressed Genes Analysis

Transcriptome assembly was performed by Trinity software (Grabherr et al., 2011). Gene function was annotated using BLAST alignment against Non-Redundant Protein Sequences (NR) and Swiss-Prot databases. Gene Ontology (GO) enrichment was implemented by GOseqR packages. Kyoto encyclopedia of genes and genomes (KEGG) pathway enrichment was carried out by KOBAS software (Mao et al., 2005). The gene expression levels were estimated between UV-B treatments and control groups by the EdgeR package (Robinson et al., 2010). The gene expression levels were estimated with RPKM (Reads Per Kilobase per Million mapped reads). The | log_2_(Treat/Control)| > 1 and the adjusted p-value < 0.005 were employed as the threshold to discriminate the DEGs.

### Phylogenetic Analysis

BLASTP alignment and HMMER program were used to identify the 2-oxoglutarate-dependent dioxygenase (2-OGD) family proteins, chalcone synthase (CHS), chalcone isomerase (CHI), flavonoid 3′-hydroxylase (F3′H), and flavonoid 3′,5′-hydroxylase (F3′,5′H) from the transcriptome data. Several representative 2-OGD, CHS, CHI, F3′H, and F3′,5′H from other land plants were retrieved from GenBank. Multiple sequence alignments were conducted using the ClustalW program. The phylogenetic tree was constructed by the neighbor-joining method using the Mega 6.0 (Tamura et al., 2013). The bootstrap values of each branch were calculated by 1000 bootstrap replicates.

### Quantitative RT-PCR Analysis

To validate the expression levels of DGEs in transcriptome Sequencing, quantitative reverse transcription-polymerase chain reaction (RT-PCR) analysis were performed. Total RNA was isolated from moss gametophytes and 0.5 ng of total RNA were used to synthesize the first-strand cDNA using the *TransScript*^®^ All-in-One First-Strand cDNA Synthesis SuperMix for qPCR with One-Step gDNA Removal Kits (Transgen, Beijing, China). The *Actin-1* gene of *L. pyriforme* was identified as the best reference gene to normalize the template. The gene specific primers were listed in Supplementary Table 1. Quantitative RT-PCR analysis was performed using *PerfectStart*^®^ Green qPCR SuperMix Kits (Transgen). The cycling regime is 95°C for 5 min, followed by 40 cycles of amplification (95°C for 10 s, 57°C for 10 s, and 72°C 10 s) and run on a LightCycler96 qPCR instrument (Roche, Switzerland). Relative gene expression levels were calculated using the comparative Ct (2^–Δ^ ^Δ^ *^Ct^*) method (Livak and Schmittgen, 2001). The experiments were carried out using three biological replicates from three different experiments.

### Metabolomic Profiling Analysis

The moss gametophytes were collected and used for LC-MS/MS analysis. The sample extraction, metabolite identification, and quantification were conducted by Wuhan Metware Biotechnology Co., Ltd. following the standard procedures (Zhou et al., 2019; Li et al., 2021; Wang et al., 2021). Briefly, the freeze-dried seedlings were ground to powder using a grinder (MM 400, Retsch, Germany). Then, 0.1 g of the powder was extracted in 1.2 mL of 70% aqueous methanol at 4°C overnight. The extract mixtures were vortexed for three times during the incubation to increase the yield. The extracts were centrifuged at 10,000 g for 10 min, and the supernatant was filtered and used for LC-MS/MS analysis. The ultra-performance liquid chromatography (UPLC) (Shim-pack UFLC CBM30A, Shimadzu, Japan) and tandem mass spectrometry (MS/MS) (SCIEX QTRAP 6500, Applied Biosystems, United States) were used. The metabolites were determined by secondary spectral properties according to the public metabolite database and the self-built database with 5000 + compounds. Principal component analysis (PCA) and orthogonal projections to latent structure-discriminant analysis (OPLS-DA) were carried out for the identified metabolites. The OPLS-DA model were used to determine the relative importance of each metabolite using a parameter called “variable importance in project” (VIP). Metabolites with fold change ≥ 2 or fold change ≤ 0.5 and VIP ≥ 1 were considered as differentially accumulated metabolites.

### Data Analysis

All experiments were performed in three biological replicates. All data were presented as the mean (± S.E.). The statistical significance of the differences between the UV-B groups and the control groups were calculated using Student’s *t*-test (*P < 0.05, ^**^P < 0.01).

## Results

### Morphological and Physiological Changes Under Ultraviolet-B Radiation

**FIGURE 2:**
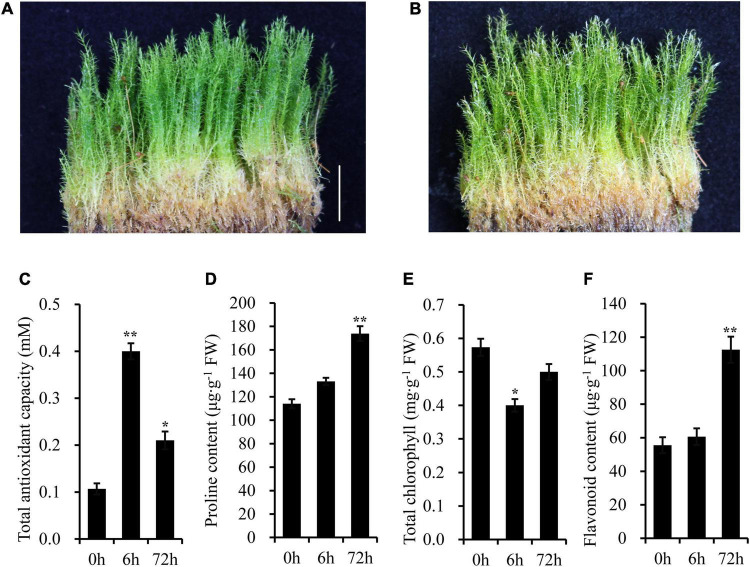
The Antarctic moss *L. pyriforme* can tolerance strong UV-B radiation. **(A)** Photo of *L. pyriforme* under nornal condition. **(B)** Morphological changes of *L. pyriforme* under UV-B radiation. **(C–F)** The physiological parameters of *L. pyriforme* under UV-B radiation. Bar = 1.0 cm.

Under normal conditions, the above-ground parts of gametophyte plants were green (Figure 2A). However, after 5 days of UV-B radiation, there was a bleaching phenomenon appeared on the top of the plant (Figure 2B). The shoots of moss also turned yellow slightly after a long-term UV-B radiation. High level of UV-B radiation produces oxygen free radicals, and further leads to oxidative stress. Then, some physiological parameters were detected after UV-B radiation (Figures 2C–F). The total antioxidant capacity was significantly higher in UV-B irradiated moss than that of the control group. For example, the total antioxidant capacity was increased to 3.75-fold after 6 h of UV-B radiation. The proline content increased gradually and reached 1.52-fold at 72 h of UV-B radiation, while the total chlorophylls in UV-B irradiated moss were markedly lower (approximately 30.23% less) than that in control group plants after 6 h UV-B radiation. Finally, total flavonoids content was increased to 2.03-fold at 72h of UV-B radiation.

### Statistical Analysis of Transcripts and Unigenes Obtained by Transcriptome Sequencing

The transcriptome sequencing was performed on an Illumina Hiseq 2500 platform (Table 1). To improve the quality and reliability of data, raw reads containing sequencing adapter or N (N represents that the base sequence is uncertain), and low-quality reads (reads with base number of Q_*phred*_ ≤ 20 accounting for more than 50% of the whole read) were removed from the original data. After filtering, the GC content distribution and the sequencing error rate were calculated, and clean reads are retrieved for subsequent analysis (Supplementary Table 2). Then, clean reads were assembled by Trinity software (Grabherr et al., 2011). The length of transcripts and cluster sequences (unigenes) were counted, respectively (Table 1). Firstly, a total of 213,691 transcripts ranging from 300 to 19,128 bp were obtained. The average length of transcripts was 1,559 bp, while the N_50_ and the N_90_ were 2,373 bp and 707 bp, respectively. Secondly, a total of 67,290 unigenes (non-redundant sequences) were assembled from the transcriptome sequencing; the statistics of length distribution showed that 24,416 unigenes were 300-500 bp, accounting for 36.28% of the total sequences, whereas 19,427 unigenes were 500-1000 bp, accounting for 28.87% of the total sequences. The average length of unigenes was 1,189 bp, the N_50_ and the N_90_ were 2,055 bp and 464 bp, respectively.

**TABLE 1:** Statistical analysis of length distribution of transcripts and unigenes.

Nucleotide length	Transcripts	Unigenes
300–500 bp	48732	24416
500–1000 bp	48920	19427
1000–2000 bp	56761	11385
>2000bp	59278	12062
Total	213691	67290
Min length (bp)	301	301
Mean length (bp)	1559	1189
Median length (bp)	1122	650
Max length (bp)	19128	19128
N_50_ length (bp)	2373	2055
N_90_ length (bp)	707	464

### Gene Functional Annotation and Gene Ontology Classification

**FIGURE 3:**
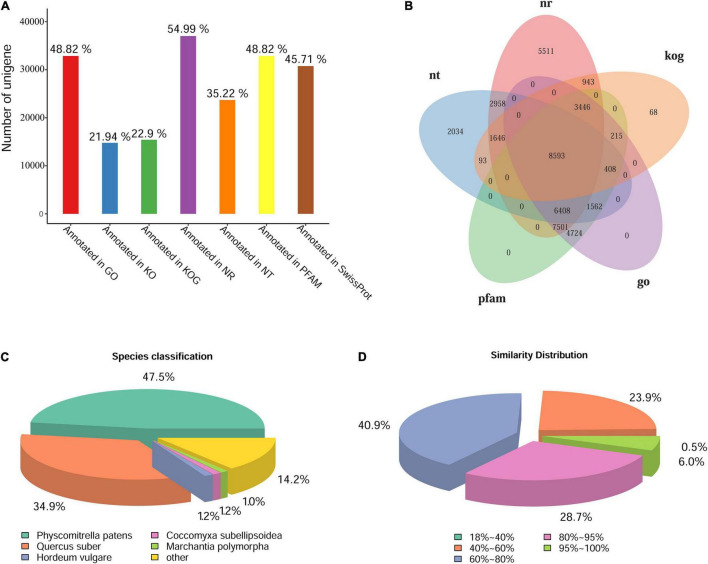
The statistics of functional annotation of the Antarctic moss genes. **(A)** The statistics of gene annotation rate in seven databases. **(B)** Venn diagram of five database annotation results. **(C)** The statistics of species classification. **(D)** The statistics of similarity distribution. Bar = 0.5 cm.

The unigenes were annotated by BLAST against the databases of GO, KOG/COG, KEGG, Nr, Nt, Pfam, Swiss-Prot. The statistics of gene annotation rate were shown in Figure 3A. There were 37,006 unigenes annotated in Nr database, accounting for 54.99% of the total, while there were 32,857 unigenes annotated in GO or Pfam, accounting for 48.82% of the total. In addition, there were 46,422 unigenes annotated in at least one Database, accounting for 68.98% of the total. Five database annotation results were selected to draw Venn diagram, and a total of 8,593 genes were annotated into these five databases (Figure 3B). According to the annotation results of Nr database, the species distribution map and sequence similarity distribution map were drawn (Figures 3C,D). The species distribution map showed the similarity between the gene sequences of this species and its related species. Five species were identified from different taxonomic positions which bore the largest number of similar proteins (Figure 3C). Results showed that *L. pyriforme* was most closely related to the model plant *Physcomitrella patens*. In addition, sequence similarity distribution analysis showed that unigenes with 60%–80% similarity comparing with the protein in Nr database accounted for 40.9% of the total, and 80%–95% similarity accounted for 28.7% of the total, respectively (Figure 3D).

**FIGURE 4:**
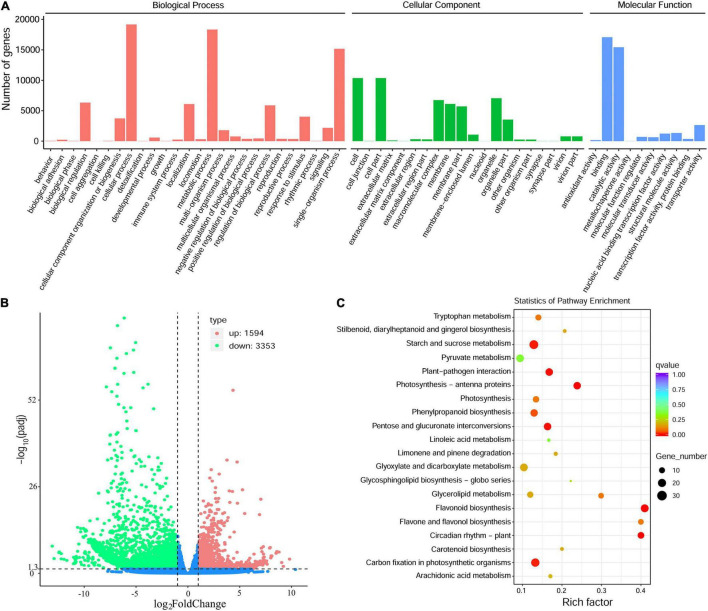
Transcriptome sequencing of the Antarctic moss under UV-B light. **(A)** The classification of Gene Ontology of *L. pyriforme* unigenes. **(B)** The volcano plot showing the DEGs between UV-B radiation group and control group. The X-axis indicates fold change of gene expression (threshold, | log_2_(Treat/Control)| > 1), while the Y-axis means the statistically significant level (threshold, q-value < 0.005). **(C)** KEGG pathway enrichment of DEGs. Rich factor represents the ratio of the number of DEGs to the total number of annotated genes in this pathway.

The annotated genes were classified according to three categories and further divided into 56 functional subgroups (Figure 4A). In biological process, these unigenes were predominantly distributed into cellular process (19,170 unigenes), metabolic process (18,337 unigenes), and single-organism process (15,172 unigenes). In cellular component, cell (10,365 unigenes) and cell part (10,365 unigenes) are the most highly enriched GO terms, followed by organelle (7,051 unigenes). In molecular function, the majority of unigenes were involved in binding (17,079 unigenes), catalytic activity (15,430 unigenes) and transporter activity (2,637 unigenes) (Figure 4A).

### Functional Analysis of Differentially Expressed Genes in *Leptobryum pyriforme* Under Ultraviolet-B Radiation

A total of 4,947 DEGs were detected by differential expression analysis using the DEGseq R package (threshold, padj < 0.05 and | log_2_FoldChange| > 1). Of them, 1,594 unigenes were up-regulated and 3353 unigenes were down-regulated in the Antarctic moss *L. pyriforme* under UV-B radiation (Figure 4B and Supplementary Table 3). KEGG pathway enrichment was analyzed by rich factor, q-value, and the number of enriched genes. Of them, Starch and sucrose metabolism, Photosynthesis-antenna proteins, Carbon fixation in photosynthetic organisms, Plant-pathogen interaction, Phenylpropanoid biosynthesis, Flavonoid biosynthesis, and Circadian rhythm were the highly enriched pathways (Figure 4C). The representative DEGs were shown in Table 2. According to their function, the DEGs were further classified into different classes involved in UV-B signaling, DNA repair, flavonoid biosynthesis, Jasmonate signaling, and ROS scavenging pathways.

**TABLE 2:** Representative UV-B stress-related genes of the Antarctic moss *L. pyriforme.*

Gene_ID	Log_2_Fold change (Treat/Control)	q-Value (p-adjusted)	Gene symbol	Functional annotation
**UV-B signaling pathway and DNA repair system**				
Cluster-30840.18892	1.25	4.36E-08	LpUVR8-1	ultraviolet-B receptor UVR8-like [*Physcomitrium patens*, XP_024386693.1]
Cluster-30840.21183	1.64	2.66E-05	LpUVR8-2	ultraviolet-B receptor UVR8-like [*Pistacia vera*, XP_031258370.1]
Cluster-30840.19346	5.70	0.00521	LpCOP1-1	E3 ubiquitin-protein ligase COP1 [*Selaginella moellendorffii*, XP_024539131.1]
Cluster-30840.14579	2.05	4.06E-08	LpCOP1-2	E3 ubiquitin-protein ligase COP1-like [*Physcomitrella patens*, XP_024371106.1]
Cluster-30840.13183	3.02	1.57E-30	LpCOP1-3	E3 ubiquitin-protein ligase COP1-like [*Physcomitrella patens*, XP_024379324.1]
Cluster-30840.1444	2.97	3.12E-23	LpCOP1-4	E3 ubiquitin-protein ligase COP1-like [*Physcomitrella patens*, XP_024371105.1]
Cluster-30840.4650	2.76	3.99E-22	LpHY5	transcription factor HY5 [*Carica papaya*, XP_021891410.1]
Cluster-30840.4633	2.26	1.12E-16	LpUVR3	(6-4) DNA photolyase [*Selaginella moellendorffii*, XP_024538612.1]
Cluster-9328.0	1.72	9.46E-06	LpPHR1	type II CPD DNA photolyase [*Pityrogramma austroamericana*, AAQ18175.1]
Cluster-20264.0	4.78	8.70E-22	LpDPL	Deoxyribodipyrimidine photolyase [*Talaromyces pinophilus*, KAF3397646.1]
Cluster-16194.0	1.86	0.00026	LpUVH1	DNA repair endonuclease UVH1-like [*Physcomitrella patens*, XP_024403241.1]
Cluster-3153.0	5.85	0.00011	LpRHP7	DNA repair protein RHP7 [*Quercus suber*, POE46986.1]
Cluster-8244.0	6.50	0.00024	LpFPG	Formamidopyrimidine-DNA glycosylase [Quercus suber, XP_023902507.1]
**Flavonoid biosynthesis pathway**				
Cluster-30840.15975	2.35	1.62E-11	LpCHS-1	chalcone synthase [*Pohlia nutans*, QBQ18373.1]
Cluster-30840.16944	2.03	1.26E-06	LpCHS-2	chalcone synthase [*Physcomitrium patens*, XP_024356740.1]
Cluster-30840.21027	1.51	0.028984	LpCHS-3	chalcone synthase [*Plagiochasma appendiculatum*, AIV42295.1]
Cluster-30840.24622	4.00	1.77E-25	LpCHS-4	chalcone synthase [*Selaginella moellendorffii*, XP_002965309.1]
Cluster-30840.17190	1.96	9.48E-11	LpCHI	chalcone isomerase [*Conocephalum conicum*, AOC83889.1]
Cluster-30840.18588	5.27	7.85E-26	LpF3H-1	flavanone 3-dioxygenase [*Physcomitrium patens*, XP_024379378.1]
Cluster-30840.4416	7.23	0.0023602	LpF3H-2	flavanone 3-dioxygenase [*Physcomitrium patens*, XP_024379378.1]
Cluster-30840.8906	1.53	2.58E-06	LpF3H-3	flavanone 3-dioxygenase [*Oryza brachyantha*, XP_006649297.2]
Cluster-30840.27891	2.11	0.00012	LpDMR6	DMR6-LIKE OXYGENASE [*Panicum virgatum*, XP_039773652.1]
Cluster-30840.26738	1.25	1.20E-18	LpF3′H-1	flavonoid 3′-monooxygenase [*Physcomitrium patens*, XP_024359127.1]
Cluster-28074.0	-2.78	0.00435	LpF3′H-2	flavonoid 3′-monooxygenase [*Vitis vinifera*, RVW52181.1]
Cluster-30840.24119	-3.95	1.84E-16	LpF3′H-3	flavonoid 3′-hydroxylase [*Raphanus sativus*, BAX90118.1]
Cluster-30840.2097	-2.37	0.00016	LpF3′,5′H-1	flavonoid 3′,5′-hydroxylase [*Pohlia nutans*, AHI15953.1]
Cluster-30840.23977	-4.88	2.59E-19	LpF3′,5′H-2	flavonoid 3′,5′-hydroxylase [*Handroanthus impetiginosus*, PIM99139.1]
Cluster-743.0	2.17	1.25E-07	LpANS-1	2-oxoglutarate-dependent dioxygenase ANS [*Physcomitrium patens*, XP_024373110.1]
Cluster-30840.16084	1.83	7.38E-09	LpANS-2	2-oxoglutarate-dependent dioxygenase ANS [*Selaginella moellendorffii*, XP_002974642.2]
Cluster-30840.19732	2.30	1.22E-07	LpANS-3	2-oxoglutarate-dependent dioxygenase ANS [*Physcomitrium patens*, XP_024374366.1]
**Jasmonic acid signaling pathway**				
Cluster-30840.18763	1.57	5.20E-05	LpOPR-1	12-oxophytodienoate reductase 3 [*Rosa chinensis*, XP_024181065.1]
Cluster-30840.15749	4.35	1.17E-55	LpOPR-2	12-oxophytodienoate reductase 11 [*Physcomitrium patens*, XP_024365650.1]
Cluster-30840.2057	1.41	2.60E-05	LpOPR-3	12-oxophytodienoic acid reductase [*Chlorella sorokiniana*, PRW44569.1]
Cluster-20359.0	3.04	4.79E-16	LpJAZ-1	jasmonate ZIM domain protein 1 [*Calohypnum plumiforme*, QTY21848.1]
Cluster-30840.16608	1.76	0.01032	LpJAZ-2	jasmonate ZIM domain protein 3 [*Calohypnum plumiforme*, QTY21850.1]
Cluster-30840.17939	2.93	5.81E-16	LpJAZ-3	Jasmonate ZIM-domain protein 9 [*Saccharum* hybrid cultivar ROC22, AVF19699.1]
Cluster-30840.2623	4.29	2.59E-25	LpRUP2	WD repeat-containing protein RUP2 [*Physcomitrella patens*, XP_024394565.1]
**Other plant abiotic stress resistance pathways**				
Cluster-30840.28529	7.94	1.87E-08	LpGST-1	glutathione S-transferase [*Klebsormidium nitens*, GAQ91203.1]
Cluster-30840.14319	2.81	8.60E-15	LpGST-2	DHAR class glutathione S-transferase [*Physcomitrella patens*, AFZ39124.1]
Cluster-30840.28471	7.88	2.88E-08	LpMAP3K	Mitogen-activated protein kinase kinase kinase A [*Symbiodinium microadriaticum*, OLP91855.1]
Cluster-30840.1781	7.04	5.51E-23	LpADH	alcohol dehydrogenase [*Quercus suber*, XP_023883167.1]
Cluster-30840.1307	3.30	0.022598	LpALDH	aldehyde dehydrogenase [*Quercus suber*, XP_023882895.1]
Cluster-20579.1	3.36	1.13E-18	LpERF-1	ethylene-responsive transcription factor ERF038-like [*Physcomitrium patens*, XP_024392192.1]
Cluster-30840.29018	2.97	4.08E-09	LpERF-2	ethylene-responsive transcription factor ERF022 [*Physcomitrium patens*, XP_024369554.1]

### Phylogenetic Analysis of Key Enzymes in Flavonoid Biosynthesis Pathway

**FIGURE 5:**
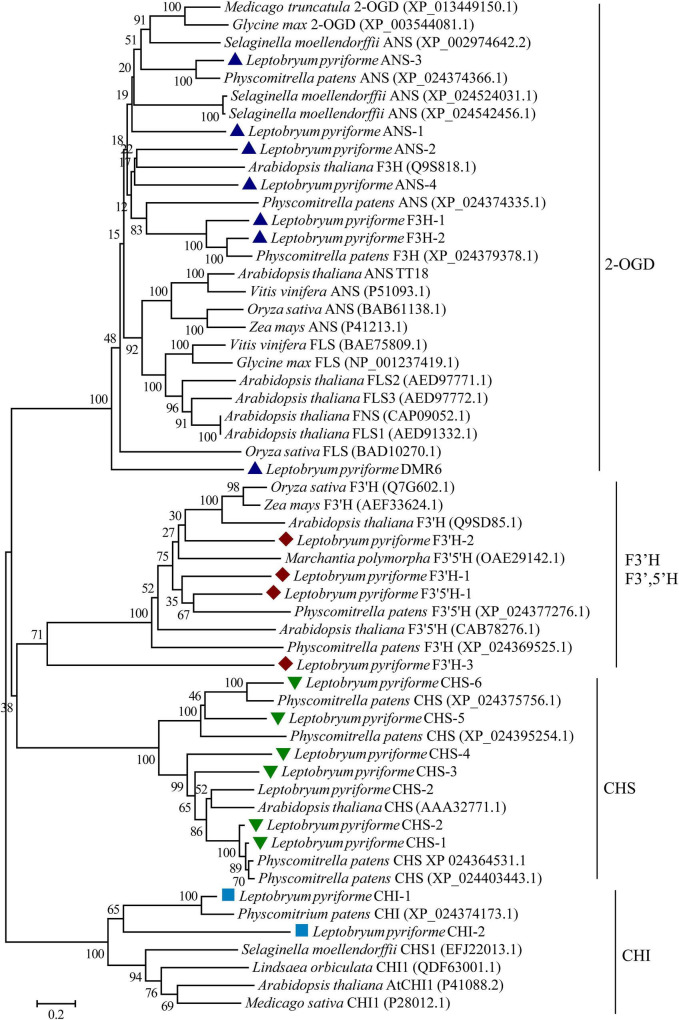
Phylogenetic relationship of flavonoid biosynthesis-related enzymes in representative plants. 2-OGD, 2-oxoglutarate-dependent dioxygenase; ANS, anthocyanidin synthase; CHS, chalcone synthase; CHI, chalcone isomerase; F3H, flavanone 3-hydroxylase; FNS, flavone synthase; FLS, flavonol synthase; F 3′H, flavonoid 3′-hydroxylase; F3′,5′H, flavonoid 3′,5′-hydroxylase.

The key enzymes involved in flavonoid biosynthesis including CHS, CHI, flavanone 3-hydroxylase (F3H), 2-oxoglutarate-dependent dioxygenase (2-ODD), flavonoid 3’-hydroxylase (F3’H), flavonoid 3’,5’-hydroxylase (F3’,5’H), were identified the Antarctic moss *L. pyriforme* transcriptome. The phylogenetic analysis of these enzymes showed that the CHS, CHI, 2-OGD were clustered together in each clade. In addition, F3’H and F3’,5’H were clustered into one clade (Figure 5). Since flavone synthase, flavonol synthase, flavanone-3-hydroxylase, and anthocyanidin synthase were 2-OGD family proteins possessing the same conserved 2OG-FeII_Oxy domain and DIOX_N domain, they formed a large tree branch. Thus, it is difficult to distinguish 2-OGD family enzymes only through phylogenetic analysis. Thus, the catalytic activities of these enzymes can be further identified by using *in vitro* enzyme activity assay.

### Metabolome Analysis of Antarctic Moss Under Ultraviolet-B Radiation

**FIGURE 6:**
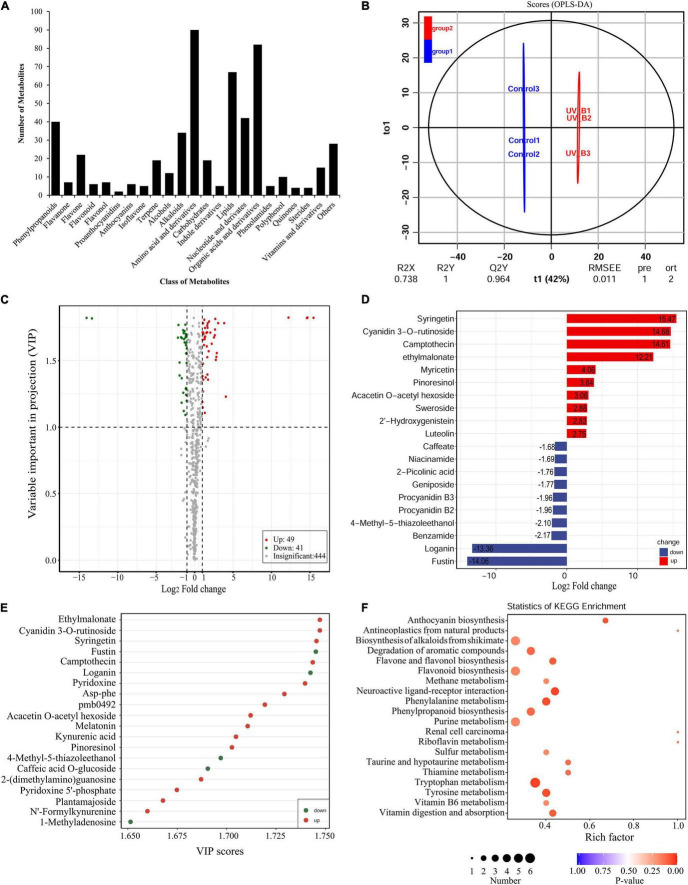
Widely targeted metabolomic analysis of the Antarctic moss under UV-B radiation. **(A)** Statistical analysis of the classes of total metabolites. **(B)** The differences between UV-B radiation group and control group were calculated using OPLS-DA model. R^2^X and R^2^Y indicate the interpretation rate of X and Y matrix, respectively. Q^2^Y represents the prediction ability of the model. A value closer to 1 means that the model is more stable and reliable. In addition, Q^2^Y > 0.5 can be regarded as an effective model, and Q^2^Y > 0.9 is an excellent model. **(C)** The volcano plot showing the contents of metabolites and the statistical significance. Each point represents a metabolite. Horizontal ordinate indicates the fold change of metabolites between two groups, while VIP value represents significant difference in statistical analysis. **(D)** The fold change of the top 20 significantly changed metabolites (SCMs) between two groups. **(E)** The VIP scores of the top 20 SCMs between two groups. **(F)** Statistics of KEGG enrichment for the SCMs.

To uncover the potential mechanisms of the Antarctic moss adapted to UV-B stress, the metabolites were detected using the UPLC-MS/MS method. A total of 531 metabolites were detected which included 90 Amino acid and derivatives, 82 Organic acids and derivatives, 67 Lipids, 42 Nucleotide and derivates, 40 Phenylpropanoids, 34 Alkaloids, 22 Flavone, 19 Carbohydrates, 19 Terpenoids, 15 Vitamins and derivatives, 12 Alcohols, 10 Polyphenol, seven Flavanone, seven Flavonol, six Anthocyanins, six Flavonoid, five Indole derivatives, five Isoflavone, five Phenolamides, four Quinones, four Sterides, two Proanthocyanidins, and 28 Others (Figure 6A and Supplementary Table 4). Therefore, Flavonoid biosynthesis pathway products including Flavone, Flavanone, Flavonol, Flavonoid, Proanthocyanidins and Anthocyanins accounted for 10.36% of the total compounds. In addition, Naringenin, Kaempferol, Quercetin, Luteolin, Eriodictyol and Hesperetin were the key intermediate metabolites of flavonoid synthesis pathway, which were all detected in the Antarctic moss *L. pyriforme*. Furthermore, Anthocyanins as the downstream products of the flavonoid pathway, were also found, including Peonidin O-hexoside, Malvidin 3-O-glucoside, Pelargonidin, Cyanidin 3-O-rutinoside, Cyanidin 3-O-galactoside, and Peonidin 3-O-glucoside chloride (Supplementary Table 4).

The OPLS-DA model was conducted to understand the overall metabolic difference between the samples in each group and the degree of variation between the samples in the group. The R^2^Y and Q^2^Y values in OPLS-DA were greater than 0.90, demonstrating that the model was meaningful, and the differential metabolites could be screened according to Fold change and the VIP value (Figure 6B). In the present study, using thresholds of | log_2_Foldchange| ≥ 1 and VIP (variable importance in project, VIP) ≥ 1, a total of 90 metabolites were identified to be significantly different in 531 metabolites after UV-B radiation. Of them, there were 49 upregulated metabolites and 41 downregulated metabolites (Figure 6C and Supplementary Table 5). Flavonoid biosynthesis pathway products accounted for 21.25% of the total differential metabolites. The top 20 SCMs in the UV-B radiation compared to the control group according to the order of | log_2_Fold change| or VIP scores, were shown in Figures 6D,E, respectively. Among them, Syringetin that belonged to Flavonols, was the most significant metabolite with log_2_(Fold change) 15.48 and VIP score 5.26. Cyanidin 3-O-rutinoside, a kind of anthocyanins, was the second significantly changed metabolite with log_2_(Fold change) 14.68 and VIP score 5.11. The significantly changed metabolites were sorted according to the type of pathways in KEGG database. These metabolites were mainly participated in the secondary metabolic pathways, such as Anthocyanin biosynthesis, Alkaloids biosynthesis, Flavone and Flavonol biosynthesis, Flavonoid biosynthesis and other small molecules metabolism (Figure 6F). Meanwhile, either DEGs or SCMs in the flavonoid biosynthesis pathway were abundantly enriched under UV-B radiation. Therefore, the changes in content of these metabolites may contribute to resisting the destruction of ROS caused by UV-B radiation.

### Ultraviolet-B Radiation Induces the Complex Network Responses

**FIGURE 7:**
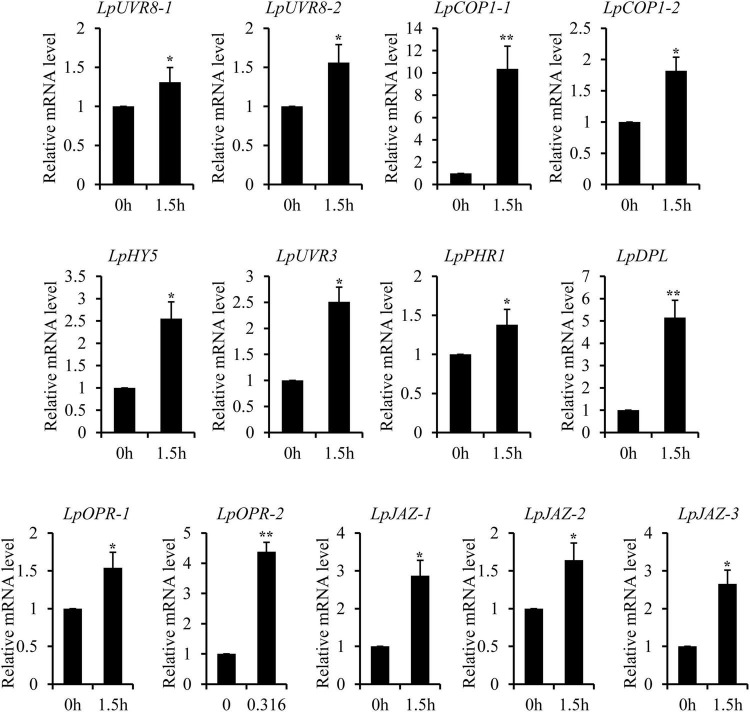
Key genes of UVR8 and Jasmonate signaling pathway were up-regulated after UV-B treatment. The gene expression levels were analyzed by quantitative RT-PCR analysis; the Y-axis indicates the relative expression level; the X-axis indicates UV-B treatment time (h); The data were calculated from three biological replicates. Vertical bars are means ± SE. Significant difference (*P < 0.05, **P < 0.01).

**FIGURE 8:**
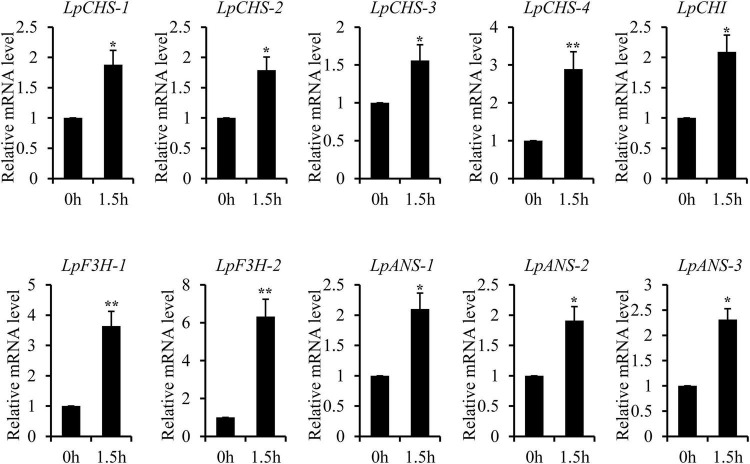
Key enzyme genes of flavonoid synthesis pathway were up-regulated after UV-B treatment. The gene expression levels were analyzed by quantitative RT-PCR analysis; the Y-axis indicates the relative expression level; the X-axis indicates UV-B treatment time (h); The data were calculated from three biological replicates. Vertical bars are means ± SE. Significant difference (*P < 0.05, **P < 0.01).

**FIGURE 9:**
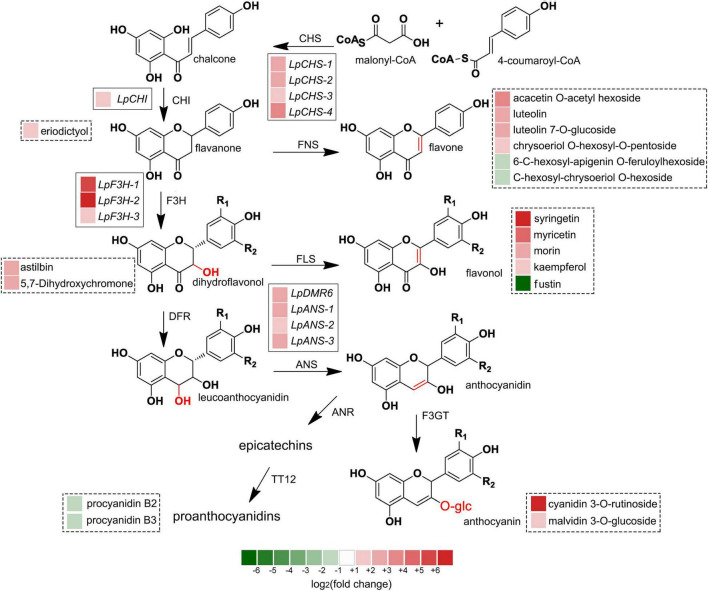
Integrated transcriptome and metabolome analysis showed that flavonoid biosynthesis might contribute the resistance of Antarctic moss against UV-B radiation. The left block of each gene and metabolite indicated the log_2_(fold change) of this gene and metabolite between control and UV-B radiation. Genes labeled in solid line box and metabolites showed in dotted box.

The recent discovery of the UV-B-specific photoreceptor UVR8 allows an in-depth evaluation of the role of downstream hormones. The DGE analysis of transcriptome sequencing showed that UVR8-COP1-HY5 signaling pathway genes were up-regulated under UV-B radiation (Table 2). Quantitative RT-PCR analysis confirmed that the gene expression levels of *LpUVR8-1*, *LpUVR8-2*, *LpCOP1-1*, *LpCOP1-2*, *LpHY5* were increased under UV-B radiation (Figure 7). Furthermore, we also found that several photolyases (i.e., *LpUVR3*, *LpPHR1* and *LpDPL*) participate in repairing the UV-induced DNA damage in a light-dependent manner, which were also up-regulated under UV-B radiation. Effects of UV-B on plants largely rely on the regulation and cross talking with hormonal pathways. In this study, JA signaling pathway-related genes (i.e., *LpOPR-1*, *LpOPR-2*, *LpJAZ-1*, *LpJAZ-2* and *LpJAZ-3*) were up-regulated under UV-B radiation (Figure 7). Jasmonates are critical signals involved in regulating the biosynthesis of secondary metabolites, especially in modulating anthocyanin accumulation. The differential expression analysis of transcriptome sequencing showed that several enzyme genes of flavonoid biosynthesis pathway, including *CHS*, *CHI*, *F3H*, *F3′H*, *F3′,5′H* and *ANS* were up-regulated under UV-B radiation (Table 2). Several flavonoid-related genes (*LpCHS-1*, *LpCHS-2*, *LpCHS-3*, *LpCHS-4*, *LpCHI*, *LpF3H-1*, *LpF3H-2*, *LpANS-1*, *LpANS-2*, *LpANS-3*) were selected and the expression levels of these genes were all up-regulated after UV-B radiation which were analyzed by real-time PCR (Figure 8). These results were consistent with the metabolome analysis data that several Flavonoids were accumulated under UV-B radiation (Supplementary Table 5). Transcriptomics integrated with metabolomics showed that the contents of flavonones (Eriodictyol, 4′,5,7-Trihydroxyflavanone, Naringenin chalcone, and Naringenin) produced by the *CHS* and *CHI* genes were slightly upregulated with the fold changes of 3.14, 1.90, 1.89, and 1.72, respectively (Figure 9 and Supplementary Table 5). Subsequently, flavones (Acacetin O-acetyl hexoside, Luteolin, Luteolin 7-O-glucoside, and Chrysoeriol O-hexosyl-O-pentoside), flavonols (Syringetin, Myricetin, Morin, and Kaempferol), and anthocyanins (Cyanidin 3-O-rutinoside, and Malvidin 3-O-glucoside), were generated under the action of 2-ODG family enzymes (FNS/FLS/ANS), which also showed significant upregulation under UV-B radiation (Figure 9). Therefore, we speculated that UV-B perception signaling, DNA repair system, Jasmonic acid signaling and Flavonoid biosynthesis pathways play the mutually coordinative roles in improving resistance against UV-B radiation in the Antarctic moss *L. pyriforme*.

## Discussion

In Antarctic, terrestrial ecosystems in particular experience harsh environments such as enhanced UV-B radiation, strong wind, less nutrient supply, less water availability, short growth season (Chown and Convey, 2016; Convey and Peck, 2019). Thus, Antarctic terrestrial plants are at the physiological limitations of survival. Consequently, bryophytes dominate the Antarctic land vegetation communities and only two vascular plants are present (Cannone et al., 2016; Bertini et al., 2021). Since 1974 the depletion of stratospheric ozone over the Antarctic has led to enhanced UV-B radiation (Rozema et al., 2005). It is therefore obvious that Antarctic terrestrial ecosystems have faced severe ozone depletion and enhanced UV-B for up to 47 years. However, only limited data on the impact of UV-B irradiance on Antarctic plants are available. In the present study, morphological and physiological analysis showed that the Antarctic moss *L. pyriforme* can endure strong UV-B radiation (Figure 2). Then, the transcriptomics and metabolomics profiling of the Antarctic moss *L. pyriforme* under UV-B radiation were detected. A total of 67,290 unigenes with the average length of 1,189 bp were generated from the transcriptome sequencing (Table 1). The differential expression analysis was widely used in transcriptome sequencing to uncover stress-related genes (Wang et al., 2021). In this research, 1,594 genes were upregulated, and 3353 genes were downregulated in *L. pyriforme* under UV-B radiation (Figure 4B). These DEGs can be further classified into pathways of UV-B signaling (e.g., ultraviolet-B receptor UVR8, E3 ubiquitin-protein ligase COP1, and transcription factor HY5) and DNA repair system [e.g., (6–4) DNA photolyase, type II CPD DNA photolyase, deoxyribodipyrimidine photolyase, DNA repair endonuclease UVH1, and formamidopyrimidine DNA glycosylase], flavonoid biosynthesis (chalcone synthase, chalcone isomerase, lavanone 3-dioxygenase, flavonoid 3′-monooxygenase and flavonoid 3′,5′-hydroxylase), jasmonic acid signaling (12-oxophytodienoate reductase 3, jasmonate ZIM domain protein, and WD repeat-containing protein RUP2) (Table 2). The moss model plant *P. patens* was found to be more capable of surviving UV-B stress than Arabidopsis and approximately 400 differential expression genes were identified from moss in response to UV-B radiation (Wolf et al., 2010). UVR8 signaling well coordinate to regulate the expression of plant nuclear genes, leading to UV-B light-induced photomorphogenesis and environmental adaptation (Podolec et al., 2021). Our results demonstrated that UV-B radiation stimulated the gene expression of UVR8 signaling components and the accumulation of flavonoids in the Antarctic moss *L. pyriforme* (Figures 6–8). In addition, transcriptional profiling of another Antarctic moss *Pohlia nutans* showed that UV-B exposure enhanced the transcript abundance for UVR8 and flavonoid pathway genes (Li et al., 2019). These suggested that the UVR8-induced flavonoid production might act as core UV-B protection mechanism and have been already established in the moss species.

Metabolomics is one omics approach of qualitatively and quantitatively analyzing all metabolites to provide a functional screen of the cellular state (Jang et al., 2018). The HPLC-MS/MS-based plant metabolomics has been widely used to profile stress-responsive metabolites (Li and Song, 2019; Wang et al., 2021). In the present study, a widely targeted metabolomics approach based on the UPLC-MS/MS analytical platforms were used to analyze the metabolites of *L. pyriforme* under UV-B radiation. Results showed that 49 metabolites were up-regulated, and 41 metabolites were down-regulated in *L. pyriforme* under UV-B radiation (Figure 6). These significantly changed metabolites were classified into metabolic pathways of Anthocyanin biosynthesis, Alkaloids biosynthesis, Flavone and flavonol biosynthesis, Flavonoid biosynthesis and other small molecules metabolism (Figure 6F). Among the 10 metabolites with the largest increases, 6 metabolites belong to flavonoids (Supplementary Table 5). Among them, syringetin (a kind of flavonol) was the most significantly changed metabolite of with log_2_Fold change 15.47 and VIP score 5.26. Syringetin was considered as one of potent anti-photoaging agents due to its UV-absorbing and antioxidant properties (Jung et al., 2016). Cyanidin 3-O-rutinoside (a kind of anthocyanin) was the second significantly changed metabolite with log_2_Fold change 14.68 and VIP score 5.11 (Supplementary Table 5). Therefore, these results provide reliable evidence for the view that Antarctic bryophytes can form an effective protective mechanism by synthesizing UV-absorbing pigments (anthocyanins and carotenoids). Reports had demonstrated that the Antarctic mosses (*Ceratodon perpureus*, *Bryum pseudotriquetrum*, *Grimmia antarctici*, *Schistidium antarctici*) and liverwort (*M. polymorpha*) contain anthocyanins, while Antarctic algae do not contain anthocyanins (Singh et al., 2011). The accumulation of flavonoids will decrease the transmittance of UV-B light (Singh et al., 2012; Singh and Singh, 2014). Furthermore, integrated transcriptome and metabolome analysis revealed that flavonoid biosynthesis may dominate the resistance of the Antarctic moss *L. pyriforme* against UV-B radiation (Figure 9).

Besides flavonoids, plants also produce structurally diverse specialized metabolites, including bioactive alkaloids. Some of them were either similar to or even more active than standard antioxidants. In the present study, three alkaloids (i.e., camptothecin, L-dencichin, and melatonin) were markedly upregulated under UV-B radiation (Supplementary Table 5). Camptothecin generally produced by *Camptotheca acuminata* and functioned as a pentacyclic quinoline alkaloid with anti-cancer activity due to its ability to inhibit DNA topoisomerase (Zhao et al., 2017). The architecture of chromatin at a given promoter is critical for triggering the transcriptional readout (Bhadouriya et al., 2020). Here, we proposed that camptothecin and modulation of DNA topoisomerase are related with DNA stabilization against UV-B-induced denaturation while maintaining its metabolic activity. Melatonin is another alkaloid compound that is upregulated under UV-B radiation. Melatonin is a multifunctional signaling molecule, ubiquitously distributed in different parts of plants (Khan et al., 2020). Melatonin acted as a powerful growth regulator and antioxidant, which delayed leaf senescence, lessened photosynthesis inhibition, and improved redox homeostasis and the antioxidant system through a direct scavenging of ROS and reactive nitrogen species (RNS) under abiotic and biotic stress conditions (Debnath et al., 2019).

Flavonoids are widely distributed metabolites in land plants. Flavonoids are synthesized through the phenylpropanoid and acetate-malonate metabolic pathways (Buer et al., 2010). They are considered to have arisen during plant evolution from aquatic to terrestrial about 500 million years ago (Davies et al., 2020; Stiller et al., 2021). However, flavonoid biosynthetic pathway and its regulatory mechanism are less characterized for bryophytes than angiosperms (Davies et al., 2020). Through the comparison of genetic and molecular studies, it is found that bryophytes and angiosperms have both commonalities and significant differences in flavonoid biosynthesis and metabolic regulation (Davies et al., 2020). The gene sequences of these enzymes have been identified from bryophytes, but *in vitro* enzymatic property analysis integrated with *in vivo* biological function analysis are recommended to identify the branch metabolic pathways. For example, at least 17 CHS genes were uncovered in *P. patens* genome, indicating that the gene family expansion and functional differentiation events occurred during the evolution of bryophytes (Koduri et al., 2010). In the present study, 6 CHS genes were identified from the Antarctic moss *L. pyriforme* transcriptome (Figure 5). The biosynthesis of flavones and flavonols requires chemical conversion of a common precursor, (2S)-flavanone, and is catalyzed by flavone synthase I (FNS I) and flavanone 3β-hydroxylases (F3Hs), respectively. Both enzymes as well as flavonol synthase (FLS) and anthocyanidin synthase (ANS) belong to the 2-OGD family proteins (Li et al., 2020; Ge et al., 2021). The functions of these enzymes in bryophytes are rarely reported. Previously, a liverwort FNSI gene was isolated from *Plagiochasma appendiculatum*, and its translating products showed efficient FNSI activity that convert naringenin to apigenin and 2-hydroxynaringenin (Han et al., 2014). The liverwort FNSIs evolved into a dual-function enzyme with both FNS I and F3H activities in both *P. patens* and *S. moellendorffii*, suggesting that they represent the functional transition forms between canonical FNSIs and F3Hs (Li et al., 2020). The sequence of key enzymes involved in flavonoid biosynthesis were also retrieved from the Antarctic moss *L. pyriforme* transcriptome (Figure 5 and Table 2). However, more equivalent functional studies need to be carried out on the basal lant plants, such as mosses and liverworts.

Mosses are restricted to sparse ice-free areas of Antarctic frozen continent. They seem to have established an efficient DNA damage repair system through synthesizing antioxidants such as UV-B-absorbing pigments and anthocyanins (Singh et al., 2011; Waterman et al., 2018). However, the analysis approaches used in previous studies only utilized the simple instrument of spectrophotometer (i.e., UV-B-absorbing compounds at AUC_280–315*nm*_, anthocyanins at A_526*nm*_) (Waterman et al., 2018). Thus, it is urgent to carry out qualitative and quantitative analyses of individual flavonoids in bryophytes. In the present study, we analyzed the metabolome of the Antarctic moss *L. pyriforme* by widely targeted metabolomics technology (Figure 6). A total of 531 metabolites were detected and flavonoids accounted for 10.36% of the total compounds. Interestingly, Anthocyanin products were also detected, including Peonidin O-hexoside, Malvidin 3-O-glucoside, Pelargonidin, Cyanidin 3-O-rutinoside, Cyanidin 3-O-galactoside, and Peonidin 3-O-glucoside chloride (Supplementary Table 4). The anthocyanins cannot be detected in methanolic extracts of *P. patens* separated by HPLC method (Wolf et al., 2010). Thus, whether bryophytes can synthesize anthocyanins is still controversial and more research are recommended to draw a conclusion. In the Antarctic field experiments, the contents of total chlorophyll were decreased in moss (*B. argenteum*) and lichen (*U. aprina*) under an enhanced UV-B radiation, while the levels of carotenoids, phenolics, and UV-B-absorbing pigments were all increased (Singh and Singh, 2014). On the plants surface, the accumulation of flavonoids will decrease the transmittance of UV-B light and have antioxidant functions (Singh et al., 2011; Dias et al., 2020). Taken together, our results suggested that UV-B signaling and DNA repair system, flavonoid biosynthesis, Jasmonate signaling pathways contribute a critical role in Antarctic moss acclimating to ozone depletion and enhanced UV-B radiation conditions.

## Data Availability Statement

The original contributions presented in the study are publicly available. This data can be found here: National Center for Biotechnology Information (NCBI) BioProject database under accession number PRJNA767045.

## Author Contributions

SL and ZZ conceived the original research, designed the experiments, and wrote the manuscript. SL, SF, and LZ performed the experiments. SL, CL, LZ, and BC analyzed the data. ZZ supervised the experiments. All authors contributed to the article and approved the submitted version.

## Conflict of Interest

The authors declare that the research was conducted in the absence of any commercial or financial relationships that could be construed as a potential conflict of interest.

## Publisher’s Note

All claims expressed in this article are solely those of the authors and do not necessarily represent those of their affiliated organizations, or those of the publisher, the editors and the reviewers. Any product that may be evaluated in this article, or claim that may be made by its manufacturer, is not guaranteed or endorsed by the publisher.
