# Supplementary material for: Transcriptomics Integrated With Metabolomics Reveal the Effects of Ultraviolet-B Radiation on Flavonoid Biosynthesis in Antarctic Moss
Source: Front Plant Sci. 2021 Dec 8;12:788377. doi: 10.3389/fpls.2021.788377 (PMC8692278; doi:10.3389/fpls.2021.788377)
Supplement: Supplementary file 1 [file Data_Sheet_1.zip › Supplementary Table 1 .DOCX]

**Supplementary Table 1** Primers used in quantitative RT-PCR analysis.

| **Gene ID** | **Gene symbol** | **Primer name** | **Primer sequence (5’-3’)** | **Annealing temperature (°C)** | **Product length (bp)** |
| --- | --- | --- | --- | --- | --- |
| Cluster-30840.18892 | UVR8-1 | LpUVR8-3F | GCGTGTGGTGACAGCCATTGT | 59.9 | 125 |
|  |  | LpUVR8-3R | GGAACCTTCTGTGGACGGAGTGA | 60.3 |  |
| Cluster-30840.21183 | UVR8-2 | LpUVR8-4F | TGGTCAGGTTGGTCAAGGCAGTA | 59.9 | 153 |
|  |  | LpUVR8-4R | TGATTCCCACCGAAGGCATACAC | 59.1 |  |
| Cluster-30840.19346 | COP1-1 | LpCOP1-1F | CGGTTCCTCCTAATTCGCCACTC | 59.5 | 154 |
|  |  | LpCOP1-1R | ACTGCTGGTGAAGGTGACAATCC | 59.2 |  |
| Cluster-30840.14579 | COP1-2 | LpCOP3-2F | TGATTGCCAGTAGCGACTATGAGG | 58.8 | 160 |
|  |  | LpCOP3-2R | ACCTTGCCATCATCACTTCCAGAG | 59.1 |  |
| Cluster-30840.4650 | HY5 | LpHY5-F | GCAGCAGACAGGCACCGATATG | 60 | 150 |
|  |  | LpHY5-R | TGCGTATGTGACAAACCAGGAACC | 60.1 |  |
| Cluster-30840.4633 | Photolyase | LpUVR3-1F | TCCTACCACCGTCATTCCAGAACTC | 60.5 | 150 |
|  |  | LpUVR3-1R | CTAACAGGGCTGAACACCTCAACAC | 60.4 |  |
| Cluster-9328.0 | Photolyase | LpPHR1-2F | GTAACGATCCATCCAAGCCAACAG | 58.5 | 152 |
|  |  | LpPHR1-2R | CACAATCAACTCCTCCAGGAATGC | 58.4 |  |
| Cluster-20264.0 | Photolyase | LpPDL-F | TTGTACGGCTGTTGGCTGATAAGG | 59.7 | 127 |
|  |  | LpPDL-4R | GCACGATACCACGGCGAATACAC | 60.6 |  |
| Cluster-30840.18763 | LpOPR1 | LpOPR1-F | AGAGTGGTGTTGGCTCCGATGA | 59.7 | 186 |
|  |  | LpOPR1-R | TTCCACCTGCTCCGTTGTGTAGA | 60 |  |
| Cluster-30840.15749 | LpOPR2 | LpOPR2-F | AGGAGCAGGTGGAAGCTTGGAAG | 60.8 | 157 |
|  |  | LpOPR2-R | GTGGTGATGCGTTGGTTGGTTGA | 60.5 |  |
| Cluster-20359.0 | LpJAZ1 | LpJAZ1-F | TCTACGCTGGCATGGTCAATGTC | 59.4 | 142 |
|  |  | LpJAZ1-R | GCTGGTGGCGGTGATGATGATG | 60.3 |  |
| Cluster-30840.16608 | LpJAZ2 | LpJAZ2-F | CCTCACTCACCTGGCAATCACG | 59.6 | 179 |
|  |  | LpJAZ2-R | TGTGCTGTGCGAGGTTGCTTAC | 60 |  |
| Cluster-30840.17939 | LpJAZ3 | LpJAZ3-F | ATGCGGAAGTCTCCAGCTCTGAT | 59.9 | 136 |
|  |  | LpJAZ3-R | CCTTGTTGCCTGTGTTGCCTTGA | 60.4 |  |
| Cluster-30840.15975 | CHS-1 | LpCHS-1F | AACGATTCTTCCTGAGAGCGATGG | 59.3 | 153 |
|  |  | LpCHS-1R | CAGTCTGGAGATCCGATGCACTTC | 59.6 |  |
| Cluster-30840.16944 | CHS-2 | LpCHS-2F | AGCTGAAGCTGAGCAAGGAGAAGA | 60.3 | 173 |
|  |  | LpCHS-2R | GAACCCGATGAAGAACCCGAACTC | 60 |  |
| Cluster-30840.21027 | CHS-3 | LpCHS-3F | GCGGAAGCAGAGAACACGGAATG | 60.7 | 157 |
|  |  | LpCHS-3R | CACTCGCCATGTTGCCATACTCC | 60.3 |  |
| Cluster-30840.24622 | CHS-4 | LpCHS-4F | AGATGTGCGGTGTGAGTGATAACG | 59.7 | 153 |
|  |  | LpCHS-4R | TTGTGAGGCTGGGTTCGGTGTAG | 60.8 |  |
| Cluster-30840.17190 | CHI-1 | LpCHI-1F | GCTGGTGCGGATCTCCATCATCA | 61.1 | 121 |
|  |  | LpCHI-1R | AGCCTCTTCTTCCTCGTCCTCGTA | 60.9 |  |
| Cluster-743.0 | ANS-1 | LpANS-1F | TCTCCATAGCTGCCTTCTCCAGTC | 60 | 169 |
|  |  | LpANS-1R | CACGCATCAGTAGTGAGAGTCTTGG | 59.8 |  |
| Cluster-30840.16084 | ANS-2 | LpANS-2F | GAATGGCGTGCGAAGCAATGAG | 59.5 | 139 |
|  |  | LpANS-2R | CTCCGACTCCGAGCGAATGAAC | 59.4 |  |
| Cluster-30840.19732 | ANS-3 | LpANS-6F | TTTCTCGCCGCAATCCCAATCG | 60.2 | 166 |
|  |  | LpANS-6R | GGCTCTCCAACTGCCTCTTCCA | 60.3 |  |
| Cluster-30840.18588 | F3H-1 | LpF3H-1F | CATACTGATCCGCACCTGCTTACC | 59.9 | 162 |
|  |  | LpF3H-1R | GCTCTTGTACCGAGTGTTGCTCAA | 59.9 |  |
| Cluster-30840.4416 | F3H-2 | LpF3H-2F | TGTCGCTGGCTTGTTTCCTGAAC | 60.2 | 140 |
|  |  | LpF3H-2F | CGGGTGGAACTTGTAGGCATTGG | 60.3 |  |
| Cluster-30840.2861 | LpActin-1 | LpActin-1F | TGTCTGGTGGCTCGACGATGTT | 60.3 | 166 |
|  |  | LpActin-1R | TGTTGGAAGGTTGAGAGCGAAGC | 59.8 |  |
| Cluster-30840.16965 | LpGAPDH | LpGAPDH-F | GGTGATTGAGGGAACGGGAGTG | 59 | 150 |
|  |  | LpGAPDH-R | TCGTGCTTGTAGTCCTGCTCGT | 60 |  |
