# Supplementary material for: Transcriptomics Integrated With Metabolomics Reveal the Effects of Ultraviolet-B Radiation on Flavonoid Biosynthesis in Antarctic Moss
Source: Front Plant Sci. 2021 Dec 8;12:788377. doi: 10.3389/fpls.2021.788377 (PMC8692278; doi:10.3389/fpls.2021.788377)
Supplement: Supplementary file 1 [file Data_Sheet_1.zip › Supplementary Table 2 .DOCX]

**Supplementary Table 2** Raw data filtering, sequencing error rate and GC content distribution.

Raw Reads, Number of reads in raw data; Clean Reads, Number of reads after raw data filtering; Error (%), Overall data sequencing error rate; Q20 (%), Percentage of bases with Phred value greater than 20 in total bases; Q30 (%), Percentage of bases with Phred value greater than 30 in total bases; GC Content (%), Percentage of G and C in four bases in clean reads.

| **Sample** | **Raw Reads** | **Clean Reads** | **Clean Bases** | **Error (%)** | **Q20 (%)** | **Q30 (%)** | **GC Content (%)** |
| --- | --- | --- | --- | --- | --- | --- | --- |
| Control_1 | 63195790 | 62356606 | 9.35G | 0.03 | 97.71 | 93.59 | 52.72 |
| Control _2 | 51601320 | 50797232 | 7.62G | 0.03 | 97.78 | 93.75 | 52.95 |
| Control _3 | 53477064 | 52750906 | 7.91G | 0.03 | 97.61 | 93.43 | 52.39 |
| UV-B_1 | 59099932 | 58502004 | 8.78G | 0.03 | 97.72 | 93.61 | 52.9 |
| UV-B_2 | 47462528 | 46998262 | 7.05G | 0.03 | 97.48 | 92.96 | 52.58 |
| UV-B_3 | 68379292 | 67434996 | 10.12G | 0.03 | 97.63 | 93.42 | 51.94 |
